# Perspectives on the cost of goods for hPSC banks for manufacture of cell therapies

**DOI:** 10.1038/s41536-022-00242-7

**Published:** 2022-09-29

**Authors:** Jung-Hyun Kim, Eihachiro Kawase, Kapil Bharti, Ohad Karnieli, Yuji Arakawa, Glyn Stacey

**Affiliations:** 1 Korea National Stem Cell Bank, Osong, Korea; 2 Division of intractable diseases research, Department of chronic diseases convergence research, Korea national institute of health, Osong, Korea; 3 Division of Clinical Basis for ES Cell Research, Center for Human ES Cell Research, Institute for Frontier Life and Medical Sciences, Kyoto University, Sakyo-ku, Kyoto, 606-8507 Japan; 4 Ocular and Stem Cell Translational Research Section, Ophthalmic Genetics and Visual Function Branch, National Eye Institute, National Institutes of Health, Bethesda, MD USA; 5 Adva Biotechnology, Hadolev3, Bar Lev Industrial Park, Israel; 6 Facility for iPS cell therapy, CiRA foundation, Kyoto, Japan; 7 International Stem Cell Banking Initiative, Barley, Herts, SG88HZ UK; 8 National Stem Cell Resource Centre, Institute of Zoology, Chinese Academy of Sciences, 100190 Beijing, China; 9 Institute for Stem Cell and Regeneration, Chinese Academy of Sciences, 100101 Beijing, China

**Keywords:** Pluripotent stem cells, Regenerative medicine

## Abstract

This report summarizes key issues contributing to the cost of preparing human pluripotent stem cell lines for use in cell therapy manufacturing based on discussion between stem cell banking experts from ten countries at a workshop session on ‘cost of goods’ for human pluripotent stem cell banking organized by the International Stem Cell Banking Initiative (ISCBI) held at the Korea National Institutes of Health in Korea (25^th^ September 2019). In this report, we also build on the workshop discussion and highlight and discuss the full range of costs and unexpected challenges on resources for the delivery of stocks of hPSCs suitable for use as starting materials in the manufacture of stem cell-based medicines. The experiences of global leaders from different national resource centers highlight issues to consider in cost management and the possibilities for reducing costs while moving into the clinical application stage.

## Introduction

The manufacture of advanced cell-based medicines is often complex and costly and prone to the unexpected impacts on the manufacturing process and this is especially relevant for products derived from cultured cells^1,2^. The strategies adopted for the expansion and processing of cells can impact significantly on the ‘cost of goods’ (COGs) for the manufacturing process and control of these costs is a crucial element to facilitate an economically viable supply chain for advanced cell-based medicines^3^. Efforts to address COGs for cell-based medicines may start with increased efficiencies in the cell culture process (e.g., culture media usage, use of better defined media to reduce variation, increased batch-size)^4^. Evaluation of cost-effective manufacturing with human pluripotent stem cells (hPSCs)^5^ as starting materials will need to include expansion of the stem cells, their processing, and testing^6^. However, whilst the broad and significant challenges which arise at the creation of these crucial starting materials have been addressed by others^7^, the variation in COGs between different sources of hPSCs in different jurisdictions have not yet been explored in detail. It is important to note that the definitions of raw materials may vary between jurisdictions and in some cases the term ‘starting materials’ is used for components that will persist in the final product such as the hPSC line used in manufacturing or vectors that are not eliminated after an iPSC line is established.

The International Stem Cell Banking Initiative (ISCBI), initiated in 2007, brings together a global community of around 300 professionals from 28 countries, including directors of the major pluripotent stem cell banks and experts in stem cell research, biobanking, regulation, and public policy development (www.iscbi.org). The ISCBI works to find consensus on issues and best practices in pluripotent stem cell biobanking and applications^7–9^. In 2019, an ISCBI workshop was held in Korea National Institutes of Health, Osong, Korea that included a session on COGs considerations for the production of hPSCs for clinical use. ISCBI experts at this workshop also discussed the management of stem cell data and genetic testing of human pluripotent stem cell lines (hPSCs) and a summary can be obtained request from admin@iscbi.org.

Key issues in COGs for cell-based manufacturing include direct costs (e.g., staff, materials services), indirect costs of organization overheads, non-recurring costs (e.g., facility construction, early product development, start-up validation), and wasted batches. Here, we report case-studies of key issues arising for COGs hPSC-biobanking in five different institutions from four countries and summarize the key points considered in workshop discussion. We then go on to identify important common issues and potential solutions relating to COGs which hPSC-based product developers should include in their considerations to manage and reduce costs. Furthermore, we identify certain impacts on costs which may not be clear at the outset of cell line development, but which may influence cell development strategies.

### Considerations for COGs with hPSCs for cell-based medicines

During the development of stem cells to the clinical stage, researchers, clinicians, and manufacturers often face unexpected costs and hurdles to bring the products to clinical trials. Workshop delegates discussed in detail the most significant cost elements and identified a number of key cost issues not always fully addressed by early product developers including:

- activities for which it may be most difficult to ascertain the cost in advance.
- the generation of cell lines that will meet regulatory requirements.
- the full requirements for construction of appropriate facilities, their validation, and maintenance.
- the demands of progression to new drug application (NDA) status.
- the additional and extensive regulatory and patient costs of clinical trials.

On the basis of these discussion topics, experts including leaders in hPSC banking, cell-based therapy product development and regulation, drew on the benefit of the combined perspectives from 27 different institutes in 10 countries represented at the workshop, to highlight issues to be included in considerations for the management of the COGs of hPSC starting materials both allogenic and “autologous”. The workshop was led by five case-studies from four countries.

### Case-studies on Banking hPSCs for Clinical Trials

#### Case Study 1: Korean National Institute of Health (KNIH) experiences in cost of goods considerations for production/quality tests of hPSCs for clinical applications, Jung-Hyun Kim (Korea National Stem Cell Bank, KNIH, Korea)

The KNIH has recently manufactured hPSCs in GMP-compliant facilities^10^ and is supplying them as starting materials in two ways: firstly, for a number of clinical applications and secondly, by partnering with other researchers and contract manufacturing organizations (CMOs) since 2018. The total area of clean rooms and the QC area of production facilities are ~1600 m^2^, and the maintenance cost of is ~$2.2 million (USD)/year). This cost does not include raw materials and product QC costs. KNIH experience is that the facility maintenance costs, such as the temperature, humidity, pressure, gas, and equipment monitoring, validation systems, and environmental monitoring for 24 h/7 d etc., are much higher than any other costs, and the percentage of the labor cost is high (Table 1). Therefore, Dr. Kim considered that reducing the ‘dead time’ of manufacturing facilities is one of the key ways to reduce costs and facility rental or use of a CMO may present a more cost-effective solution than the option for a stem cell bank to build and run its own facilities.

**Table 1:** An example of the KNIH’s costs (approximate value).

Direct cost (PD, QC, QA)	MSC (1 lot, 160 vials)	iPSC (1 lot, 130 vials)
Labor fee (per year)	240,000 (USD)	240,000 USD(USD)
Materials fee (per lot)	115,000 (USD)	9000 (USD)
QC (outplant testing and in house)	33,000 (USD)	58,000 (USD)
In-direct cost (per year)		
Equipment qualification and requalification (fee for service)	333,000	
Environmental monitoring	Total 250,000 (Including labor fee 100,000 USD)	
Hygiene and building sanitation	Total 250,000 (Including labor fee 100,000)	
Facility operating costs	Total 1,500,000 (Including labor fee 525,000)	

It is important to note that GMP licensing as approved by national authorized bodies, applies to the manufactured product and includes assessment of the suitability of all aspects of manufacturing including, but not limited to staff, procedures, materials, and facilities. The manufacture of the production cell lines as starting materials may not be considered part of manufacturing process, but they are typically assessed for suitability by regulators. Raw materials are often prepared under international standards for medical devices such as ISO 13485 (see ISCBI meeting report available from admin@iscbi.org).

Other significant costs were associated with obtaining all the necessary documentation and traceability for donor cells, but KNIH had not yet identified generic methods to minimize the associated costs as the ease of obtaining adequate documentation varies considerably depending on the source of donated cells. However, it is important to select and fully document donor cells to ensure that they will meet the applicable regulatory requirements.

In addition to these specific issues a number of learning points which enabled KNIH to address cost reduction in development of iPSC lines for clinical application were shared.

A standardized and well-controlled manufacturing process will reduce the unexpected ‘dead time’ of cleanroom manufacturing facilities. The manufacturing scale of production is established from lab-scale production methods and thus, there are numerous hurdles and risks which may not be feasible to predict. However, it is vital to address the challenge of developing a scale-up system that will enable production to clinically relevant cell numbers and quality. Having achieved this, KNIH has experienced a further challenge of transferring the developed technologies into a GMP manufacturing quality management system and the requirement to translate the method into the hands of a Contract Manufacturing Organization (CMO). The latter issue in particular adds a further and significant level of complexity. Translation time for academic protocols for use in KNIH facilities had varied depending on product type, but had typically taken a minimum of 3 months. Difficulties and delays at this translational stage are very costly and the CMO staff typically require specialist training in the cell culture process. In the KNIH experience of iPSC generation the development of lab-scale processing using culture-ware and raw materials that are known to be suitable for GMP manufacture, has shortened the technology transfer time as it was not necessary to adapt the cultures to new reagents during the product development phase.

Donor eligibility tests conducted to find suitable donors may take time, which can lead to unexpected delays in manufacturing, however, formal donor selection criteria can help to make this process more efficient^7^. Finding suitable donors for cells with a defined homozygous HLA type can be challenging^11,12^. In such cases, cell sources from the cord blood banks would be useful to find homozygous lines, however, their utility depends on the suitability of donor selection procedures used^13^. Nevertheless, staff time must still be allocated to carefully review donor traceability, documentation of donor eligibility testing and contents of the individual informed consents.

Contacting the regulatory authority at an early stage, certainly before commitment of significant resources on manufacturing facilities and the product development stage, has significantly helped KNIH to avoid delays to clinical application. For example, the regulatory authority is likely to request full and documented traceability, including the certificates of origin and certificates of analysis (CoA) of all raw- and starting-materials including the Sendai virus that is used for iPSC generation. If the iPSC lines have been generated where such traceability was not established, it will be much more difficult to demonstrate suitability of the iPSC to the regulator’s satisfaction and KNIH has found that frequent discussion with the Korean regulatory authority is important before starting the manufacturing process in a GMP facility to avoid the need for costly changes to procedures and even facility adaptation, before manufacturing can commence.

Qualification of the seed stock or master cell bank (MCB) has been an important step for assurance of KNIH product manufacturing. KNIH staff have focused on performance and documentation of critical quality control (QC) including cell line identity and adventitious agent testing of the MCB. This has reduced the risk of product failure and can limit delays and unexpected costs by ensuring MCB QC information is sufficient prior to initiation of the GMP manufacturing process. Currently, Korea’s Regulatory Authority (MFDS) requires, amongst other testing, the submission of the results of short tandem repeat (STR) tests of the seed stock (primary cells) for both the MCB and working cell bank (WCB) for manufactured cell therapy products.

Human errors in cell therapy product manufacturing and GMP-compliant seed-stock banking processes often lead to extra costs and delay product outcomes (e.g., misreading of SOPs, mis-recording of data, failure to maintain aseptic technique, use of incorrect reagents). Although such errors may be rare, they can have serious consequences, such as discarding of product batches, closure, and emergency clean-down of production areas, followed by requalification of facilities. KNIH has therefore focused on avoidance of the likelihood of human errors by ensuring that written instructions and procedures are clear and use unambiguous language. However, even more crucial is staff training which is mandatory under Korean national regulations, and only qualified staff can be involved in the manufacturing process. Natural disasters such as violent weather systems and earth-quakes can damage the integrity of facilities and back-up systems. Human viral epidemics and pandemics can also threaten adequate staffing and have incurred additional unexpected costs which have had to be managed by KNIH on a number of occasions. Electrical power failure may well cause shut-down during the manufacturing process leading to very high unexpected costs because product may be lost and instruments and environmental controls may need to be requalified and disinfected prior to restarting manufacturing processes. Therefore, risk assessment, implementing of risk management plans and establishing disaster recovery procedures are recommended by KNIH in order to prepare for expected natural disasters. Such procedures will also help to protect manufacturing from other kinds of risk to the raw materials supply chain and product safety and quality.

#### Case study 2: experiences from a development of an automated CART manufacturing process, Ohad Karnielli (Avda Biotechnology Ltd, Israel)

Dr. Ohad Karnielli described attempts to make mesenchymal stromal cell (MSC) manufacture more efficient using a semi-automated bioreactor with automated real time monitoring of glucose levels^14^. In addition, Dr. Karnielli reported on a new automated and controlled CAR- T platform which can deal with the autologous manufacturing challenge. He presented a case study in which a facility wished to manufacture 20,000 doses a year. This challenge required a platform capacity to carry out 500 manipulations per day under clean room conditions for 720 patients in parallel with 55 new patients per day. Only an automated and fully controlled platform can enable such capacity and in turn assure the quality.

Dr. Karnielli went on to describe the translation costs from R&D to GMP. Compared with the R&D development time, translation to GMP manufacturing had taken around 3x longer which meant costs for developing a GMP process were $3M minimum in the presented case. Translation of the process for GMP manufacture to a CMO could then take ~9 months and the uncertainty of the time-lines for these aspects increases costs. Cleanroom consumables could be a major cost element for cell culture processed and in Adva’s experience could represent 50% of total manufacturing costs.

#### Case study 3: Eihachiro Kawase (Institute for Frontier Life and Medical Sciences, University of Kyoto, Japan)

Dr Eihachiro Kawase pointed out that it was important to consider future projected need for the number of vials and cells per vial required, and costs of validation including preservation and storage. Failure to address these aspects could lead to the necessity for repeated banking campaigns, causing delays and additional production and testing costs.

At the stage of clinical studies, the development costs for the product implantation method and also for patient monitoring and financial reimbursement systems for implementation warranty could be very significant and need to be considered.

Most significant costs experienced in Dr. Kawase’s hESC manufacturing facility in Kyoto had been:

- Cell culture related consumables and notably media suitable for manufacture of clinical products (StemFit AK03N, Ajinomoto, Japan), matrix (iMatrix-511MG, Matrixome, Japan), and ROCKi (Y-27632, Fuji Film Wako, Japan).
- Outsourced testing including biological safety evaluation including endotoxin testing, pathogenic microorganism contamination tests and antibiotic residues testing (NB. currently, Dr. Kawase’s group spend about $ 10K/lot (i.e. for each cell bank)).
- Staff costs including training which is especially challenging under Japanese law (i.e. the “5 year rule”), can make it difficult to retain long-serving employees.

It had been difficult to accurately cost QC, validation, and ICH Q5A requirements for testing, however, no significant costs had emerged that had not been expected. A significant cost over time in Japanese industry　had also been the requirement for maintenance fees to get governmental permission, as in Japan a special cell processing facilities were required to be replaced every 5 years.

#### Case study 4: generation of patient bespoke iPSC for manufacture of retinal pigmented epithelium, Dr. Kapil Bharti (National Eye Institute, NIH, USA)

Dr. Kapil Bharti had found that the largest cost in hPSC-derived RPE was the time in GMP manufacturing and he believed that anything to reduce that would improve COGs. For Dr. Bharti’s group, contracting out GMP manufacture had proven more costly than in-house provision. Validation was also a major part of the cost but QC, whilst not cheap, was not their major cost. Dr. Bharti summarized key NEI-NIH costs in the RPE manufacturing and some possible cost reductions as follows:

- Patient viral testing: $500/patient (in-house).
- Plasmid for reprogramming: $2000 per round of reprogramming (Sendai vectors <$1500).
- Media and reagents: $25–30 K (bulk purchase important to keep costs down).
- Karyology: $450/clone.
- Proving plasmid loss from each cell line: $1000/clone (this could be controlled to <$250 using Nanodrop technology).
- Oncogene screen: $1600/clone (use of non-CLIA regulated testing could keep costs to below $500)
- STR analysis: $220/clone.
- Flow cytometry: $200/clone (in-house).
- Sterility assays: $200–500/clone (in-house).

He reflected on certain costs of patient bespoke (i.e., “autologous”) iPSCs that were difficult to calculate including manufacturing cell lines at a rate of only one or two at a time in early trial phases. This requires maintenance of all staff even when there is limited demand and whilst it was anticipated that this would become more consistent at fuller capacity operation, it would only be realized when moving into phase III clinical trials.

Dr. Bharti also discussed the costs of this kind of “autologous” versus allogenic approach. Autologous lines required replicated banking and testing costs for each cell line compared to a single process for an allogenic product. However, there were some benefits for autologous cells that included:

- Cost saving where adventitious agent testing had not been required for autologous donors.
- Limited cell passaging was needed for autologous cells compared to allogenic product which required cells to be expanded to greater scale for multiple patients.

Dr. Bharti explained that the current challenges for the NEI-NIH facility were in obtaining sufficient GMP cleanroom occupancy and down-time for maintenance which was a crucial issue causing delays and increased cost. He also described possible means to reduce this for example by making maintenance more efficient and less invasive and running multiple manufacturing batches together in the same facility. The latter possibility could be particularly beneficial, but would require validation. Such parallel (i.e., non-campaign) manufacturing would require procedural and physical protection mechanisms to keep products separate will be essential to meet GMP requirements.

Another way Dr. Bharti envisaged streamlining production was to make quality system SOPs consistent for different products where possible and at the NIH facility there were 25 common protocols used for different patient products.

A solution also considered beneficial by Dr. Bharti for reduced cost was to rent cleanroom space and avoid long-term costs re dead-time when there is no work to do and down-time required for maintenance.

#### Case study 5: COGS experiences at the CiRA iPSC center, Yuji Arakawa (Facility for iPS cell therapy, CiRA foundation)

Dr. Yuji Arakawa reported that manpower costs were ~50% of total cost in the CiRA facility and other significant costs were due to quality assurance (QA), maintenance, and servicing. These can be high per banking activity if outsourced, but in-house provision meant ongoing continuous costs. CiRA also found that outsourced virus testing and whole genome sequencing were costly. Cell banks were produced at 300 vials per bank which took 1 month to produce and cost $300,000 overall per batch. Dr. Arakawa noted that different sources of iPSCs varied in charge per vial and some charges varied depending on the intended use. He reported that the CiRA foundation was planning to sell iPSC stocks at $1000/vial. This was broadly consistent with other hPSC banking centers such as Wicell (www.wicell.org), UK Stem Cell Bank (www.ukscb.org), and EBISC (www.ebisc.eu) that supply cell lines in the range of $1000 to $1500 depending on the application, however, some other banking centers such as the stem cell facility at the Korea National Institute of Health provide cell lines at no charge.

### Workshop open discussion

The sharing of industry-ready cell manufacturing protocols and cost efficiency measures were concluded to be important to enable cost-efficient uptake of cells established for manufacturing purposes.

One challenging issue was assuring that new automated bioreactors would be acceptable in GMP manufacturing and in particular from the perspective of the potential for contamination. Delegates concurred that experience indicated that supplier claims of “closed” culture systems needed to be validated carefully. It was noted that importance of validation not only related to facilities and equipment, but also to implementation of new analytical methods, such as multiplexed PCR primer systems, which often required method adaptation and thus additional validation. Delegates agreed that facility and equipment validation was a significant burden in both time and resource in one case (personal communication, Dr. Hashimoto, DS Pharma, Kobe) the balance of cost of validation across manufacturing was as follows: 45% staff, 23–25% testing and 25% materials.

The discussion also turned to the issue of false claims of “GMP” quality of equipment. This had also been covered in the discussion at previous ISCBI meetings and had been a clear concern for regulators (see notes from ISCBI Los Angeles June 2020 available on request from admin@iscbi.org).

Delegates reflected on the fact that molecular interventions in manufacturing can also raise complexities regarding safety and quality control. The development of gene-edited hPSCs for therapeutics was also likely to incur additional costs for quality controls which may include as yet to be determined safety testing as already discussed in ongoing ISCBI workshops (see ISCBI workshop proceedings, Melbourne June 2018, summary available on request from admin@iscbi.org). Furthermore, the preparation of cell substrates may have impacts for downstream costs as experienced for CART cells where the viral load (i.e. copy number per cell) may need to be monitored in patients post treatment.

Costs for supply of vials of cells for use in manufacture were discussed for different centers and it was clear that as discussed in the case studies, there was significant variation from $1000 to 10,000 per vial and in some cases special contract conditions were understood to incur additional charges depending on factors such as stage of product development. Delegates also agreed that the cost assessment for cells intended for future manufacture should also include implementation of clinical laboratory standards. These would now include the recently established standard for clinical testing ISO15189^15^, that will be particularly important given the increasing implementation of new analytical methods for cell-based medicines including genome-wide clinical genetic testing.

## Discussion and conclusions

COGs is a crucial element in enabling uptake of advanced hPSC-based medicines or cell therapies^6^. The history of animal cell biotechnology has shown that translation from lab scale research procedures to manufacturing is not simply a matter of increasing the size of the culture system. Many factors in the cell culture system which affect cell growth such as changes in culture vessel materials, different liquid/atmosphere surface areas for gas exchange, sheer stress from bubbles or impellers and altered mass or heat transfer dynamics in the culture medium^16–18^, all potentially impact on the efficiency of large-scale systems compared to the researcher’s original protocols. In addition, the biomarkers and analytical approaches may need to be developed during product development^19,20^ so that it is difficult to calculate cost of goods for manufacturing on the basis of a research protocol yet to be translated into a GMP manufacturing process. Furthermore, the comparison of COGs in different national and institutional settings is challenging and figures for the cost of production of hPSC lines has been difficult to assess^21^. However, in this paper experiences from a number of centers making hPSCs for manufacturing cell-based medicines have consistently indicated that the key direct and most obvious manufacturing costs arose from the need for experienced staff, use of qualified clean-room facilities, specialist cell culture materials and testing for quality control and safety. One example of autologous CART manufacture has shown that the largest component of labor costs could be over 70% of the total manufacturing cost^22^, comprising mostly manufacturing processing (48%), quality control (16%), and quality assurance (16%). In this case materials were the next highest COGs element (18%) and costs of facilities (8%) and equipment (4%) seemed to represent a relatively minor element of the COGs. it is vitally important too. However, another study of a different T-cell adoptive therapy differed in that facility costs were highest (56% of total), with labor and materials both significantly less (i.e. ~20% each)^23^. It is important to recognize that these costs arise within the manufacture of an autologous cell product with bioprocessing far less complex than for an hPSC-derivative. In one comparison of an adoptive T-cell therapy product and hPSC COGs for each appeared to have a similar profile. the COGs for the hPSC system simply assessed costs for development of a master cell bank of an hPSC line (i.e. a starting material) and not an actual product^23^. However, this study calculated average costs for very early and late stages of preclinical development and revealed that both in the case of the hPSC bank and adoptive T cell therapy, facility costs decreased compared to labor costs as each process progressed towards clinical trial stage. Thus, the ability to directly translate labor costs and COGs in general, from reports of one product type to another and between somatic cell and pluripotent stem cell manufacturing are not straightforward even where the products are nominally very similar. For manufacturing of hPSC-based products the cell culture systems are multifaceted, generate complex mixed cell populations, are often much more prolonged (up to 300 days for batch cultures of certain hPSC derivatives) and also based on complex raw materials often not available under appropriately qualified conditions. For the hPSC banking process alone, whilst the challenge of creating a reproducible cell differentiation process is not an issue, the stability of the system during cell expansion and qualification of novel raw materials and assays can still create high COGs.

Validation of the manufacturing process was also considered a major and necessary cost, but one which was often not obvious to early-stage product developers. As commented on by Dr. Bharti (NEI-NIH) (Case study 3 above), validation procedures for GMP manufacturing and could cost almost as much as the item being validated i.e., cost of validation of vector removal in some cases had exceeded the cost of vector manufacture. Indeed, other areas of cell manufacture such as CART therapy facility validation costs in might make up 20% of the total facility costs^22^, but again translation of such COGs elements between somatic and pluripotent stem cell manufacturing processes can be problematic for the reasons already stated. Furthermore, COGs evaluation between regional jurisdictions may also vary depending on the predicted equipment and facility life-span (which may be prescribed in national law as illustrated in case study number 3 by Dr. Kawase) and the specific frequency of facility and equipment requalification required. Validation is also required for various aspects of the manufacturing process and includes, but is not limited to, facilities (including environmental monitoring), reagents, equipment, analytical methods, storage, and shipment. Thus, early assurance that raw materials, vectors and production cell lines would all meet regulatory requirements was crucial to avoid costly changes at a later stage. Finally, the importance and costs of qualification of the cell source (e.g., donor selection, consent, bio-sampling, storage) will be important to consider, especially if multiple donors are required as in haplo-banking or generation of autologous cell lines. The ISCBI workshop speakers and delegates also considered opportunities where the direct costs could be cut and concluded that avoiding commitment to ongoing manufacturing facilities by rental of clean room space or outsourcing manufacture to a CMO was attractive. However, this could also increase timelines and incur new costs due to the need to either wait for access to manufacturing slots and/or train CMO staff. Also, in relation to efficient use of cleanroom time the need to plan and prospectively manage down-time for maintenance was considered important to avoid costs due to unplanned down-time. A number of workshop presentations reflected on the use of CMOs or facility rental and some of the key pros and cons to be considered are summarized as described in Table 2. In making decisions it is important to clearly understand the long-term goals of the biobanking operation although actual future developments can be extremely difficult to predict due to changes in funding, new scientific developments, and new institutional strategies.

**Table 2:** A comparison of in-house banking, facility rental, and CMO options for hPSC biobanking and control of COGs.

Considerations	In-house biobanking	Facility rental	Use of CMO
Advantages	Maintain control over know-how (staff training and development, protocols, intellectual property (IP) etc.), quality management, and facility access. In theory this enables greater flexibility and smoother progression).	Fee per service can reduce costs of in-house set up and maintenance, enable quicker start up times and will not affect or be affected by, other institutional activities.	Sponsor institute does not have to build appropriate facilities or recruit regulatory, QA, and manufacturing staff. Manufacture can run unaffected by sponsor institution activity
Disadvantages	long lead-in time and high cost to build and staff the facility. Ongoing costs of facility maintenance, regulatory compliance, and ultimately facility replacement after ~20years (or as little as 5 years as required in Japan).	Core staff may need to be taken away from regular duties to run the facility or recruit and train dedicated staff which also adds to cost. Need to align quality management systems and operational aspects of the facility provider and product developer. Access may need to be negotiated years in advance.	Risk of an unsuccessful and costly technology translation process requires time commitment for careful preparation of documentation, reagent supply pipelines, and comparability between CMO and product developer processes. Potential loss of know-how and potential IP due to technology transfer to CMO. Loss of control of the manufacturing process.
Crucial consideration	The ongoing costs of maintaining the facility and staff should be considered as part of production life cycle and longer-term development plans.	The manufacturing process should be worked out completely prior to transfer to the rental facility to avoid extended rental periods increased.	The manufacturing process should be worked out completely prior to transfer to avoid high CMO costs and potential loss of in-house IP.

Approaches used under GMP to address this include planned preventative maintenance (i.e., replace key parts before failure and enable better production campaign planning) and design features that enable maintenance from outside of the cleanroom without compromising cleanroom integrity and air quality. Examples typically used in GMP manufacturing include ceiling or wall embedded fittings permitting maintenance without lab entry and gas supplies piped and filtered through the cleanroom wall.

Processing of multiple cell lines or products in the same cleanroom at the same time were also considered attractive to enhance cost efficiencies in terms of campaign progress, staff time, and reducing cleanroom occupancy time. However, this approach would also require rigorous process controls to assure separation of different cultures and products to avoid switching of process materials and cross-contamination.

Outsourced testing continues to be a cost which is difficult to reduce. Care is needed to assure the quality of testing and reporting and a key prerequisite should be use of testing services subject to formal accreditation by appropriate professional bodies. Attempts to carry out in-house testing, while bringing timelines within greater control of the manufacturer, could also incur significant staff-time and training commitment. In addition, they represent an additional quality assurance burden (e.g., staff time, staff expertize, equipment, and validation). The decision to out-source versus in-house provision will be influenced by a number of local factors including ease with which the staff can accommodate the testing, level of quality assurance support and availability of specialist equipment and facilities. Testing which is carried out for information and is not used for cell bank or product release^7^ is typically most readily accommodated in-house.

Automation was an area where considerable benefit is perceived for the future with positive experience in the manufacture of medical devices^24,25^. Sealed (“closed”) automation systems offer both advantages of avoiding the need for high air quality cleanroom conditions and the limitations of ‘isolator’ operation. Much of the technology for aseptic connection of these devices for media supply, quality control sampling, and harvesting have been developed in industry production systems for cell culture-based vaccine and biotherapeutics. Challenges for automation may include unexpected reaction of stem cells to new environments and surfaces, altered mass transfer effects (i.e. variation in chemical and gas concentrations within culture medium) within the bioreactors. Microcarriers and suspension systems are well advanced for manufacture of cell culture derived vaccines and biotherapeutics and are under development for culture and differentiation of pluripotent stem cells^26–28^. Furthermore, an IMI (Innovative Medicine Initiatives) - a partnership between the European Union (represented by the European Commission) and the European pharmaceutical industry (represented by EFPIA, the European Federation of Pharmaceutical Industries and Associations) - a funded hPSC-biobanking project (EBiSC, www.ebisc.org) is using a 4 × 50 ml volume stand-alone bioreactor system for biobanking cell expansion and differentiation a funded hPSC-biobanking project (EBiSC, www.ebisc.org) is using a 50-ml volume stand-alone bioreactor system for biobanking cell expansion with cells in suspension or on alginate beads^29^. Automation systems may also have the benefit of permitting reduction in size of equipment and thus, reducing the highly expensive manufacturing space needed. In cell-based medicines the use of standard commercially available culture expansion units such as described by Dr. Karnielli (see case studies above) also provide ready scalability without the significant technology transfer and validation costs often associated with the switch to full-scale manufacturing^30^. Thus, combined isolator and automated culture could in principle help to optimize cost reduction. Isolator systems for standard culture modalities had been tried in many labs and had become popular. They can reduce facility costs of a cleanroom but still require the same consumables, significant staff time and quality control costs.

It is also important not to forget the potential impact of automated quality control and substantial improvements have already been experienced for QC of autologous CART therapies (e.g., 80% timesaving with automated flow cytometry analysis) and in molecular analysis^31^. Beyond quality control methods, the overall efficiency of quality assurance can in principle^32^ and in practice be transformed by streamlining and switching to electronic operating systems.

Typically, the cell banking process involves preparation of a large master cell bank (MCB) and then following completion of quality control and characterization, a single MCB vial is thawed and re-expanded to generate a working cell bank (WCB). The WCB is then subject to a focused set of quality control tests. However, the operational and quality control costs can be made in adjusting the fundamental approach to cell banking which might be termed a “continuous banking” process. This involves retaining some cultured cells from the batch of cultures used for a MCB and expanding them immediately without cryopreservation to the WCB passage level. This avoids time consumed in recovery of cryopreserved MCB cells and potential loss of cells and increased risk of expansion of genetic variants. However, it also means that WCB may be created before the MCB testing is completed so potentially the cost of WCB could be wasted if cells at MCB level do not pass QC. However, implementation of rapid screening for genetic stability could enable early abandonment of WCB manufacture due to the most likely QC problem of expansion of genetic variants. This approach has been tested in a number of centers including the UK Stem Cell Bank (UKSCB) and has been published by the Hadassah Medical Centre^33^. It addresses a key element of COGs reduction for cell therapies which is to optimize utility of capacity^22^.

A further element that is crucial to avoiding wasted resources on failed batches is the implementation of a due diligence process during procurement of stem cell lines^7,34^^,^ This involves careful screening of candidate manufacturing cell lines and/or donor tissues to ensure the cells have the appropriate ethical provenance and biological characteristics, are free from evident microbial contamination and allow the user freedom to act without adverse intellectual property issues.

Another means to reduce early commitment of resources to hPSCs is to generate a large MCB and allocate a certain proportion of vials as an early Distribution Cell Bank. Such a system has been used at the UKSCB to substantially cut early investment in cell culture (staff, consumables, cleanroom costs) and quality control (staff time, consumables, outsourced testing), when popularity of individual hPSC lines has yet to be determined and thus overtime, allows the biobank to focus resources on those lines that become more popular with biobank users. The KNIH case-study above reflected on the significant costs that may be associated with donor procurement and selection and this is often forgotten in discussions about costs of biobanking as it was typically a historical activity which did not involve the biobank or is not such a key issue for suitability as in the case of “autologous” iPSC lines used by the KNIH and NIH facilities. However, as biobanks move towards development of cell lines specifically intended for manufacture of cell-based medicines^35,36^ and particularly where bespoke genotypes are required such as homozygous HLA haplotypes^11,12^, this is an issue that will significantly impact on the cost of generating hPSC-derived medicines. In order to reduce such costs, it is wise to consider using existing donor selection systems such as health services or sharing resources with collaborators with similar needs. In conclusion, a significant number of iPSC and hESC lines are available from professional hPSC biobanks established to assure all the correct procedures for donor selection have been documented and these make a valuable first point of contact for hPSC-based product developers seeking suitable production cell lines^7^.

Consideration of most costly elements such as facilities, services, and staff, depend significantly on the local methods for measuring costs. Many centers in academic institutions may only figure in additional costs of consumables, external testing etc. and do not include core staff, general use equipment and facilities (such as cleanroom construction and animal facilities) provided for other projects and overheads, which can be very different between different institutions or academia and industry. All of the latter items may not be included in published estimations of cost per vial or bank of cells and so need to be born in mind when comparing published cost of goods for use of hPSC lines.

In order to proactively tackle the issue of COGs reduction it is also an important consideration to identify the various sources of influence on COGs and score the impact that these might have overall. This can enable issues with more significant potential to raise costs to be prioritized and appropriate action taken to limit or reduce COGs. Some important examples of these variables in the hPSC manufacturing chain, including cell sourcing and banking, are given in Table 3.

**Table 3:** Some key variables in hPSC cell banking and manufacturing.

Stage	Variables influencing CoGs	Potential for impact on CoGs (high, moderate and low) and causes
Raw and starting materials (cell culture media, reagents, cell lines, and vectors)	Quality of risk assessment procedures Standards adopted by suppliers Degree of documentation available on sourcing materials	Depends on complexity, reproducibility: Biological origin potentially high impact Chemically defined and manufactured under industry standards typically lowest impact
Expansion of hPSC	Reproducibility of cell product from a given scale up system Change in cells which are hazardous or otherwise deleterious (loss of function, cancerous transformation)	High potential impact due to waste of total production runs due to compromised or unsafe product
Differentiation of hPSC	As given for expansion above. Reproducibility of purity and different cellular process contaminants Time-course consistency in manufacturing process to achieve bulk product specification	High potential impact due to variable composition, timing and quality/safety of product
Harvest Purification Formulation	Decline in viability Nature of cell loss (apoptosis, necrosis, autophagocytosis) Nature of excipients including cryoprotectants Container size and cell number	Moderate to High potential impact as may require increased cell input to allow for cell losses in processing.
Banking, analytical testing and other services.	The options and impact of in-house or outsourced banking activity has been described and addressed in Table 2.	Potentially High impact as described in Table 2
Shipment/timing with patient	Cryopreserved product: consistency of cooling, storage and thawing conditions Normothermic (10–20°C)^37^ product: temperature stability and time of shipment	Low-moderate impact of storage, but potentially high impact due to significant cell losses during cooling and thawing High potential impact due to biological activity (degradation, growth) or contamination during transport.

In conclusion, figures quoted in the literature for costs of hPSC lines are influenced by many factors. We have identified some that represent significant costs common to most centers making hPSCs for manufacture of cell-based medicines. However, other factors are significantly variable depending on the nature of the stem cell bank’s host organization including its core objectives, the expertize and services available in-house and how the organization manages facility costs and especially how it establishes overhead costs. Furthermore, there are costs which are less obvious to early developers (e.g., quality assurance, validation studies, and facility maintenance), and others that are much less predictable including donor recruitment and technology transfer where CMOs are used for manufacturing. Any organization wishing to derive its own hPSC production cell lines, should consider the utility of existing resources such as those engaged in the ISCBI community (www.iscbi.org) who are supplying hPSCs specifically for manufacture of cell-based medicines. These centers will have already addressed the key issues for assuring the suitability of their hPSC stocks and can save significant investment of time and resources for the early stage of product development. Furthermore, the attention applied by stem cell resource centers to assure the suitability of cell lines^7^ will reduce the risks of failure in product development.
